# Restoration Ecology: Two-Sex Dynamics and Cost Minimization

**DOI:** 10.1371/journal.pone.0077332

**Published:** 2013-10-28

**Authors:** Ferenc Molnár, Christina Caragine, Thomas Caraco, Gyorgy Korniss

**Affiliations:** 1 Department of Physics, Applied Physics, and Astronomy, Rensselaer Polytechnic Institute, Troy, New York, United States of America; 2 Department of Biological Sciences, University at Albany, Albany, New York, United States of America; Universidad Carlos III de Madrid, Spain

## Abstract

We model a spatially detailed, two-sex population dynamics, to study the cost of ecological restoration. We assume that cost is proportional to the number of individuals introduced into a large habitat. We treat dispersal as homogeneous diffusion in a one-dimensional reaction-diffusion system. The local population dynamics depends on sex ratio at birth, and allows mortality rates to differ between sexes. Furthermore, local density dependence induces a strong Allee effect, implying that the initial population must be sufficiently large to avert rapid extinction. We address three different initial spatial distributions for the introduced individuals; for each we minimize the associated cost, constrained by the requirement that the species must be restored throughout the habitat. First, we consider spatially inhomogeneous, unstable stationary solutions of the model’s equations as plausible candidates for small restoration cost. Second, we use numerical simulations to find the smallest rectangular cluster, enclosing a spatially homogeneous population density, that minimizes the cost of assured restoration. Finally, by employing simulated annealing, we minimize restoration cost among all possible initial spatial distributions of females and males. For biased sex ratios, or for a significant between-sex difference in mortality, we find that sex-specific spatial distributions minimize the cost. But as long as the sex ratio maximizes the local equilibrium density for given mortality rates, a common homogeneous distribution for both sexes that spans a critical distance yields a similarly low cost.

## Introduction

Ecological restoration aims to replenish an ecosystem’s biodiversity, often responding to human-induced losses of indigenous species [1], [2]. When ecosystem managers reintroduce a species to its former habitat, the restoration effort’s success is ordinarily defined by combined ecological and economic criteria [3]. Similarly, optimizing biological-control programs may integrate impact on the target species with costs of deploying the control agent [4].

Consider an example where restoration failed. Historical records indicate that Canada lynx (*Lynx canadensis*) were found in New York State (NYS), but were seen only rarely during most of the 20th century [5]. Between 1989 and 1992, no fewer than 80 lynx were captured in Canada and released in the Adirondack Mountains of NYS. Each animal carried a radio-collar, so that survival and dispersal could be monitored. The lynx rapidly dispersed; mortality during dispersal was high. Lynx population density grew too low for successful reproduction, and the species is now considered extirpated in NYS [5].

Generalizing the example, we envision restoration of a single species whose population dynamics depends on the density of each sex. Before evaluating costs, we must identify those spatial distributions of the initial population that assure successful restoration. Suppose that we initiate restoration with a single spatial cluster, within which individuals are distributed at a uniform density. Then we must find the “critical cluster” size, the minimal area the species must occupy to sustain positive population growth. Analysis of the critical-cluster criterion has advanced understanding of spatial systems in both physics [6]–[8] and ecology [9]–[12]. However, if ecosystem managers can vary initial densities according to location, non-uniform spatial distributions might reduce restoration cost. Given multiple initial population distributions assuring sustained population increase, the most preferred option should *minimize* cost. Our study investigates how the minimum cost of successful restoration depends directly on spatial pattern, and how the optimal pattern depends on sex ratio, and on sex-specific mortality rates.

In this context, we model a species’ restoration as a spatially detailed, one-dimensional, two-sex reaction-diffusion system. We optimize the initial densities and spatial distributions of the sexes to minimize the cost of restoring the species to its positive, stable (homogeneous) equilibrium density throughout a habitat. The prototype of such models is the Fisher-Kolmogorov equation [13], [14]

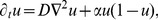
(1)which describes the dynamics of single-sex populations with logistic growth and diffusive dispersal. Our model extends the basic reaction-diffusion framework to include sex-structured dynamics [15]–[17], where an Allee effect [18], [19] generates an unstable fixed point between extinction and the habitat’s carrying capacity.

In many natural and managed populations, *per-capitum* growth is reduced as density becomes small; this is termed an Allee effect [20]–[22]. Different behavioral, ecological and genetic mechanisms can induce an Allee effect [23]. Low population density may diminish individual reproduction by reducing mate encounters, making prey capture more difficult, or by leaving individuals more susceptible to their own predators [24], [25]. Allee effects will be amplified if dispersal into unoccupied habitat reduces local population density; a sufficiently high dispersal rate can generate negative population growth, thwarting restoration [18].

Generically, the cost of restoration can be can defined as:
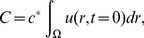
(2)where 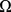 represents the extent of the habitat, and 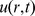 is the population density at location 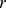. Without loss of generality, we can consider a constant *per-capitum* cost (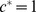). Mathematically, the restoration cost, which we seek to minimize, is a functional of the initial population’s spatial distribution. The constraint requiring population persistence cannot be expressed analytically, since that would require solving the model’s partial differential equations exactly. Therefore, we develop a population dynamics with simple processes and easily interpretable parameters, and obtain the minimum cost with analytical and numerical techniques.

We organize the rest of the paper as follows. First, we introduce our sex-structured population dynamics, and outline the analytic and numerical methods we employ in this paper. In the Results section we conduct a systematic study of the restoration cost in multiple stages, considering different initial spatial population distributions in each stage. Starting with a simplified, single-sex version of our model, we derive an unstable, aperiodic stationary solution for the PDE, and use it as initial distribution, resulting in a single-sex cost. We then continue with the sex-structured model and analyze the way restoration cost depends on model parameters, given a simple, homogeneous initial distribution of individuals inside a cluster. We refer to this initial setup as “rectangular”, for its shape on a density *vs* location plot. We analyze this setup first with the constraint of equal cluster sizes for both sexes; later we relax this constraint. In the third stage we allow any possible shape of initial population distribution and study how the cost can be reduced as a result. Finally, in the Discussion we compare the minimum costs found in each stage, and conclude which approach yields the most economical restoration.

## Methods

### General Assumptions

Our model’s key parameters include the sex ratio at birth and sex-specific mortality rates. We assume that females and males disperse independently by homogeneous diffusion, and that males encounter females as a mass-action process, equivalent to random mating [26]. The fraction of matings leading to successful reproduction is proportional to the unoccupied fraction of the environment, 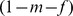 [15]–[17]. That is, the population grows in a self-regulated manner. Hence, we have:
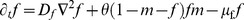


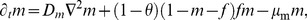
(3)where 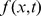 and 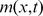 denote the local densities of females and males, respectively. Diffusion rates are described by coefficient 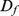 for females and 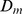 for males. Three parameters characterize the local dynamics: 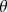, 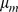, and 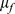, which denote the fraction of individuals born female, and density-independent mortality rates for males and females, respectively. Note that while Eqs. (3) do incorporate spatial effects, they retain a “mean-field” character (the statistical physics terminology) in that correlation functions of the underlying stochastic individual-based model are factorized into products of densities [27], [28]. In principle, by extending the above deterministic reaction-diffusion equations with appropriate noise terms [29]–[31], the resulting stochastic partial differential (or Langevin-type) equations [32] can capture the relevant macroscopic features of the underlying spatial, stochastic individual-based model [33]–[35]. (Note, however, that the rigorous derivation of such stochastic partial differential equations can be rather challenging [36]–[38]).

Our model [Eq. (3)], with spatial detail and diffusion removed, is identical to that studied by Tainaka et al. [15]. In our earlier work [17], we briefly analyzed the above two-sex dynamics with diffusive dispersal, and found the critical radius (hence, critical cluster size) for an initial population’s successful invasion of a two-dimensional habitat. While our reaction-diffusion model includes numerous simplifications for detailed application to particular species, nevertheless it exhibits the essential ecological characteristics of more complex two-sex models. Hence, implications of our results for restoration will likely hold across a wide range of specific models.

We can transform Eq. (3) to interpret it as a single-sex model by making the equations symmetric. To do so, we must let 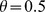, restrict 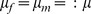, 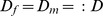 and use the same initial-density distributions for both males and females. In this way, the two densities behave identically over time: 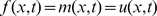, described by the following equation:
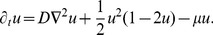
(4)


This transformation bridges the single-sex and two-sex models; using the constants in Eq. (4), we can directly compare results between models without rescaling parameters. Note that the (cubic) local dynamics also retains an Allee effect.

### Analytic and Numerical Methods

Our first approach to cost minimization selects suitable unstable stationary solutions of the PDE model as initial population distributions, since we can derive them analytically for the single-sex model. These are special “critical” solutions, which transform to stable equilibrium solutions (either persistence or extinction) depending on a small perturbation. The cost associated with each stationary solution is found by numerical integration of the density profile (i.e., the area under the curve). We obtain the stationary solutions for the single-sex model by setting the left-hand side of Eq. (4) to zero, and deriving a relationship between the stationary solution’s local value and its derivative.

For the sex-structured model, we cannot derive stationary solutions analytically. Instead, we base our study on the model’s critical limits of cluster size and density, which are directly related to the minimum cost of successful restoration. For a rectangular initial setup (homogeneous initial spatial distribution inside the cluster with a specific population density), there exists a critical cluster size, the smallest spatial extent such that the given density achieves sustained positive growth. Symmetrically, for a given cluster size, there is a critical initial density, the lowest density assuring sustained population growth. In both cases, the critical limit also corresponds to the minimum cost, since the cost is proportional to both cluster size and density. The exact, parameter-dependent values of these critical limits cannot be derived analytically. Instead, we use binary search across a range of possible values, accomplished by testing each value for successful restoration *vs* extinction.

We discriminate restoration from extinction by numerically integrating the model until it has converged to a global equilibrium. We use second-order finite difference discretization for spatial derivatives and explicit Euler method for integration over time [39], with a sufficiently small time-step. Integration stopped when all time-derivatives at all spatial coordinates were less than 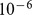. Finally, the cost is obtained by multiplying the initial density and the initial cluster size, and summed for males and females.

Next, we extend the rectangular initial setup approach by allowing the initial cluster size to differ between males and females. In this case, we first calculate the critical cluster size for males at a fixed cluster size for females using binary search, resulting in a cost with respect to the given female cluster size. Then, we use gradient descent on this cost function to minimize it with respect to female cluster size. Note, that during the gradient descent we always change the female cluster size by one unit of spatial grid resolution, and we keep moving toward the negative gradient even if the local derivative is zero, because a small slope discretized with any grid can result in zero local gradients before reaching the actual minimum point. Note also, that we strongly rely on the convexity of the cost function with respect to male and female cluster sizes.

Finally, we relax all constraints on the shape of the initial population distribution; we optimize the spatially discretized shape for lowest cost under the constraint of successful restoration. Discretization is essential because it allows us to express the cost as an 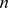-dimensional function, instead of a functional, where 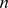 is the size of the spatial grid. We use the same grid for shape discretization and numerical PDE integration, for practical reasons. Given the discretization, we use *simulated annealing* [40] for optimizing the shape function. This is essentially a Monte Carlo simulation, where random changes of the initial spatial distribution (the shape function) are accepted or rejected according to a specified acceptance probability function, such that the visited cost states have a Boltzmann-distribution characterized by a temperature-like parameter. As this parameter is lowered, the expected value of the cost is also lowered, eventually leading to the globally optimal, minimum cost state. For the specific steps of simulated annealing, see Supporting Information (Section S5 in File S1). In order to determine whether the constraint of successful restoration is satisfied we must numerically integrate the model at every Monte Carlo step. To reduce computational time, we accelerated the PDE integration with GPGPU computation using CUDA [41], [42], which locates the global equilibrium of the PDE in a fraction of a second, giving a total time for the simulated annealing on the order of a few hours.

## Results

The prerequisite for analyzing the cost of restoration is to ensure the local stability of successful restoration, i.e., a positive stable fixed point of local dynamics. The necessary stability condition for the two-sex model [Eq. (3)] is given in our previous work [17]:
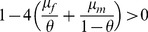
(5)


Similarily, we can find the necessary stability condition for the single-sex model [Eq. (4)]:
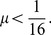
(6)


These conditions provide us guidelines for selecting proper model parameters when evaluating costs in the following sections. Justification for Eqs. (5) and (6), and formulas for the fixed points are presented in the Supporting Information (Sections S1 and S2 in File S1).

### Unstable Stationary Solutions

An unstable stationary solution of the PDE seems a good candidate for the initial spatial distribution. It is a critical solution, in the sense that given a small perturbation, the unstable stationary solution transforms to a stable, spatially homogeneous solution. Positive perturbations result in positive homogeneous population densities (successful restoration), and negative perturbations result in zero densities (extinction).

Partial differential equations, such as Eqs. (3) and (4) have infinitely many unstable stationary solutions. We cannot find these solutions directly, but we can derive analytical formulas for the relationship between the density and its spatial derivative. For the single-sex model [Eq. (4)] we have the following definitions:
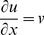
(7)

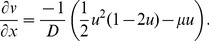
(8)


Using 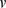, we change variables to write a first-order differential equation:
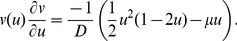
(9)


**Figure 1:**
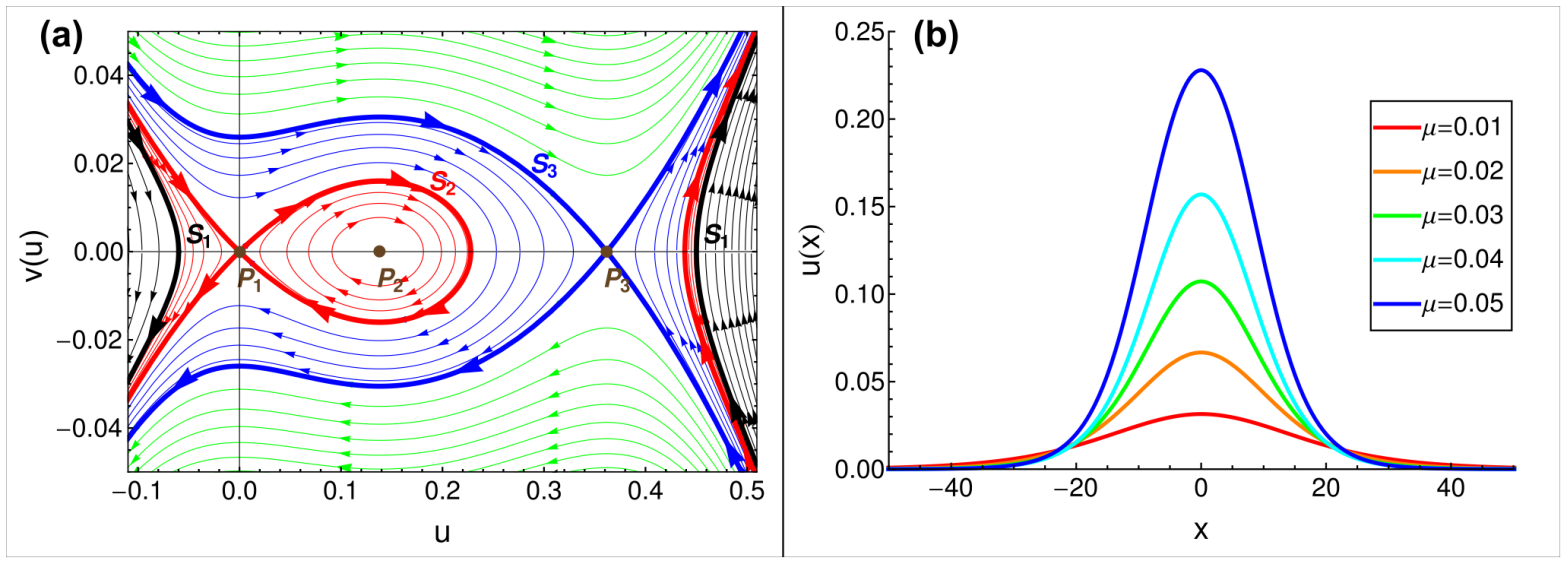
Stationary solutions in the single-sex model. (a) Phase plot of stationary solutions, described by Eq. (10). 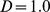, 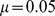. The dots indicate fixed points, the thick lines indicate separatrices. Different curves correspond to different 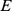 parameters, however, values of 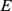 were not chosen uniformly, for aesthetic reasons. (b) The stationary solutions found by integrating along the 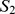 separatrix, for multiple mortality rate parameters; 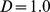; 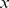 is distance form the habitat’s center.

By separating variables, we obtain the following analytical solution:
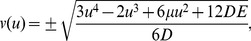
(10)where 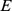 is a free parameter. The phase diagram for this equation is depicted in Fig. 1(a) (also in Fig. S2 in File S1), and its contents are summarized as follows.

The fixed points 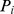 correspond to *homogeneous* stationary solutions. Naturally, these are also fixed points of the original equations [Eq. (4)], but here they are only special cases of stationary solutions that do not vary spatially. Hence 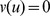. 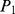 and 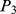 are saddle points (stable equilibrium nodes of the local dynamics) corresponding to extinction (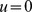) and persistence (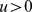), respectively. 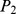 is a center (unstable fixed point of the local dynamics) corresponding to the unstable equilibrium due to the local dynamics’ strong Allee effect.

Curves in Fig. 1(a) correspond to *inhomogeneous* stationary solutions that can be classified by the value of the free parameter 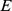 in Eq. (10). Separatrices 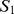, 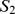, and 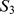 correspond to the following values (with the same subscripts, see Section S3 in File S1 for details):
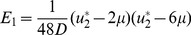
(11)

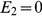
(12)

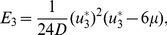
(13)where 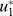, 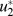, and 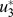 are the equilibrium densities of local dynamics, i.e., the values of 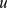 corresponding to 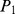, 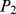, and 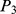, respectively:



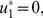
(14)

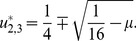
(15)


The closed elliptical curves around 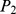 represent periodic stationary solutions, and they are the only ones of interest, because all other curves extend to infinitely large negative or positive densities, neither having biological meaning.

Spatially periodic stationary solutions may offer candidate initial population distributions. In principle, if minimum densities within each period were close to zero, then we could select a segment of the solution, one period in length between two density minima, and apply it as an initial spatial distribution. However, in our case, as the minimum value of 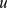 goes to zero, the period of the solutions goes to infinity, and the curves converge to the 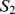 separatrix, which corresponds to an aperiodic stationary solution. The exact aperiodic shape of 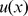 can be found by numerical integration along 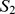, depicted in Fig. 1(b). Since 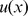 converges to zero rapidly, we can use it as an initial population distribution by taking its central segment above an arbitrary small (biologically meaningful) density threshold. Because of the fast convergence to zero, the length of the segment will be finite. In our study, we use 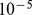 for the density threshold. Then, for every 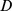 and 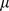 combination, we have an exact shape, and an exact cost value defined as twice the area under the curve (counting both males and females), denoted by 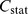. We will compare these costs with those found by the other two methods described in the following subsections.

As an interesting observation, we note that contrary to intuition, the period length of the stationary solution does not converge to zero as the solution curves approach 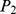, see Fig. S3 and further details in Supporting Information (Section S4 in File S1).

For the sex-structured model [Eq. 3] we cannot derive unstable stationary solutions analytically. Instead, we study the scaling of restoration cost through a numerical analysis of the critical cluster size.

### Critical Cluster Size and Minimum Cost

Criticality of an initial cluster’s size occurs when density reduction due to dispersal exactly balances the net effect of local natality and mortality; Video S1 clearly shows this form of criticality in one dimension. In two dimensions the expanding population front’s speed is reduced in proportion to the curvature of the cluster; therefore, it affects the critical cluster size. To avoid confusing effects of curvature with other parameters’ impact on the critical cluster size and, hence, the minimum cost, we restrict our study to one dimension.

We begin our analysis of the critical cluster size by assuming a rectangular initial setup. This is the most obvious choice, for its mathematical simplicity, and its plausible application (*e.g*., an animal population surrounded by a fence before release can be modeled with a uniform spatial distribution). Therefore, we have four parameters describing the distribution: 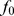, 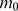, 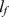, 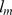, which represent female density, male density, length of space occupied by females, and length of space occupied by males, respectively, at the initiation of restoration. (Both female and male clusters are centered symmetrically). We shall refer to 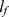 and 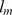 as the cluster sizes of the initial population. The cost of the initial state is defined simply as:
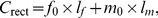
(16)and it is minimized by using the critical cluster sizes 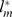 and 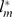, for males and females, respectively. Note, that 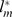 and 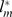 are themselves dependent on initial densities 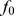 and 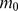, as well as model parameters 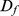, 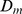, 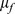, 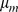, and 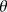. Therefore, we systematically study dependence of critical clusters on all parameters.

As an initial step, we analyze the dependence of critical cluster size on the diffusion coefficients. We anticipate that the critical cluster size, *i.e*., critical length is proportional to the square root of the diffusion coefficient. Similar scaling has been observed in two-dimensions when a population with a strong Allee effect disperses by diffusion [17], [18]. Our aim here is to show the same behavior in the one-dimensional reaction-diffusion system, and to ask whether it holds when male and female diffusion coefficients and cluster sizes differ.

**Figure 2:**
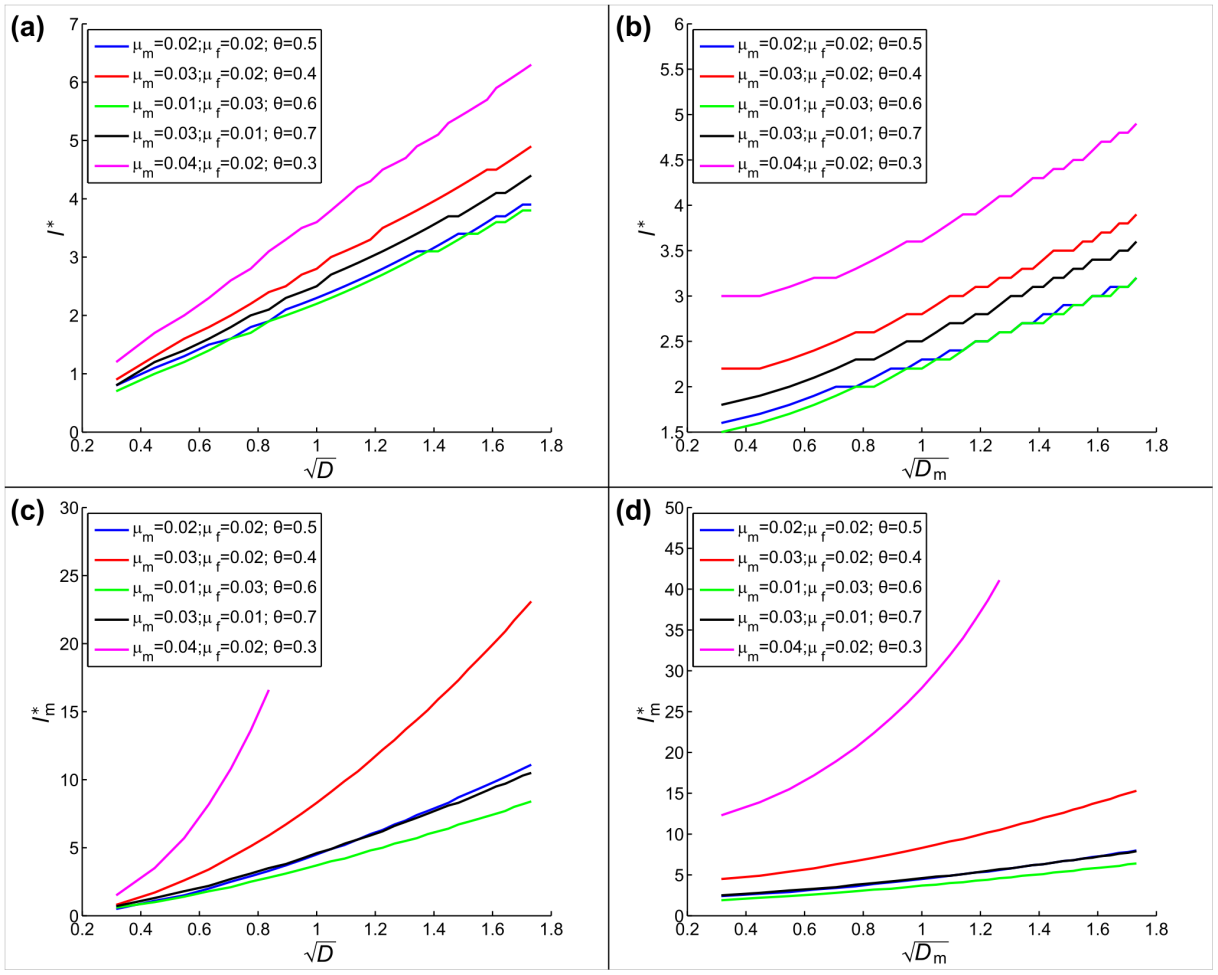
Scaling of critical cluster sizes *vs* diffusion coefficients, at various parameter values. (a) 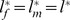, 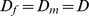; (b) 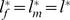, 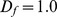; (c) 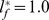, 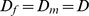; (d) 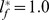, 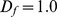. In every case, initial (spatially homogeneous) densities are fixed: 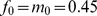.

Figure 2(a,b) shows that as long as we employ the same cluster size for males and females, the critical cluster size has the expected scaling behavior 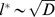 [18] with respect to both male and female diffusion coefficients, even if one sex has a fixed diffusion coefficient. Note that it is sufficient to study the dependence on the diffusion of one sex (here, males) while the other is fixed (here, females), because of the symmetric construction of the model. Further, Fig. 2(c,d) indicates that fixing the cluster size of one sex while measuring the critical cluster size of the other sex with respect to diffusion coefficients yields non-trivial scaling. However, we observe that a higher dispersal rate results in a larger critical cluster size, and, hence, a larger restoration cost.

Continuing our analysis of critical cluster sizes, we now assume that the critical cluster sizes are equal for both sexes (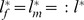); we relax this constraint later. Even with the equal cluster-size constraint, initial densities for males and females within that cluster may, in principle, differ. In practice, a density difference could be implemented for most diecious species. To find the best choice of initial density values (with respect to minimizing cost), we aim to relate them to model parameters, taken as given for the focal population.

**Figure 3:**
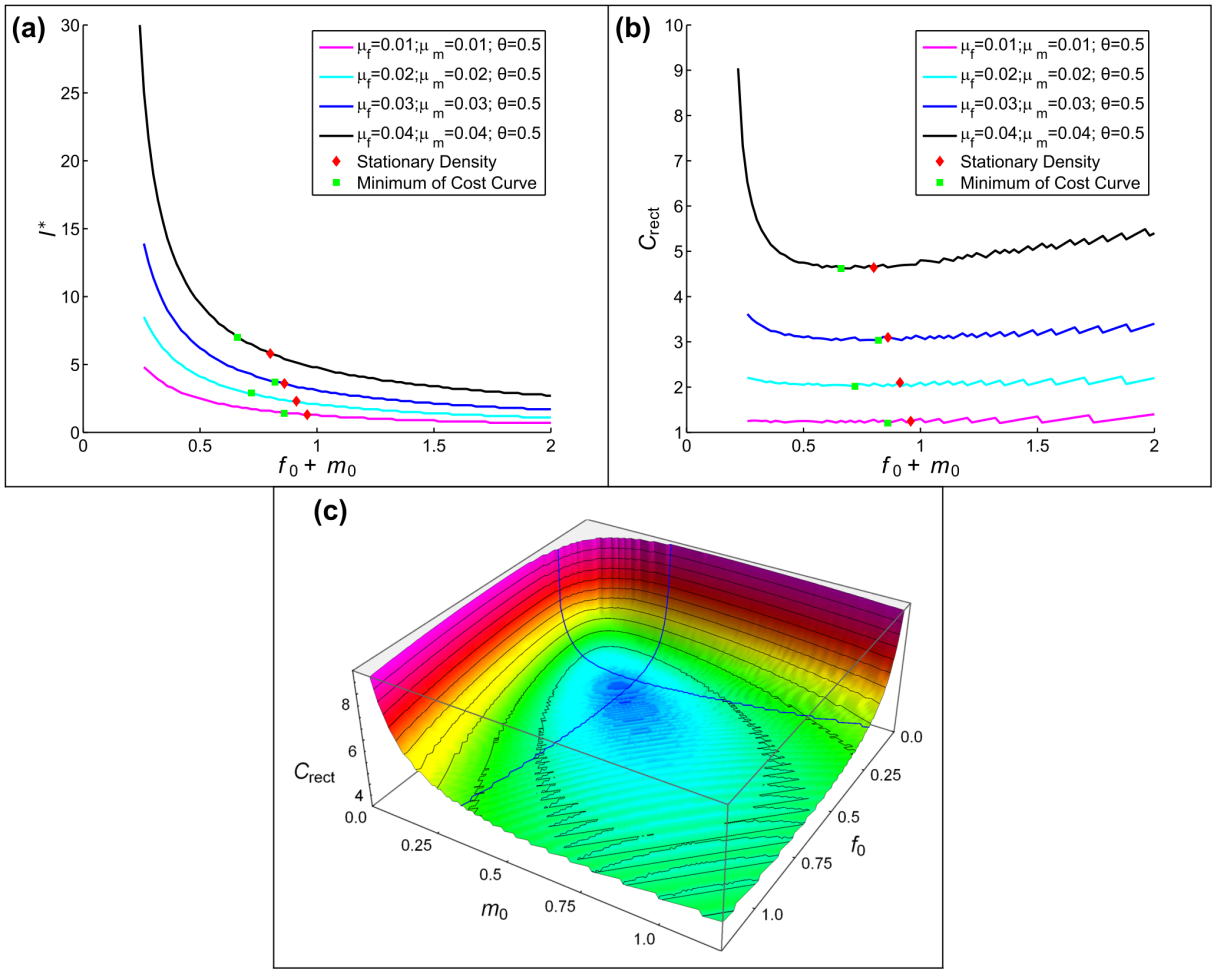
Scaling of (a) critical cluster length at introduction, and (b) cost, with respect to initial population density. The total density shown on the 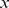 axis is divided equally between males and females. The point markers on the curves show the stable stationary densities of local dynamics. (c) shows the cost landscape with respect to male and female densities; the blue cross marks the stable stationary density values; 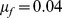, 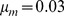, 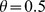. For all figures, 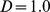.

Naturally, the initial densities must exceed the Allee threshold (the unstable fixed-point densities); otherwise, the population can never achieve positive growth. Figure 3(a) shows that as the initial density is lowered, the critical cluster size increases, and goes to infinity as we approach the Allee threshold.

Scaling of the cost, however, is non-trivial. We can always find the minimum cost at a density somewhere in the vicinity of the positive stable fixed point of the system, but always slightly below it. We understand this by considering the dynamics just after initial introduction. If the population starts from its stationary density (the stable, positive fixed point), then the local densities can only decrease, due to diffusive dispersal. However, if the initial density is lowered slightly, then the population has a chance to grow locally (in particular, at the center of the cluster) before the effects of diffusion reach it, while the eventual spread through the habitat remains the same. In essence, the cost is slightly lowered by handing over some of the spreading effort to growth dynamics. However, as Fig. 3(b) and Fig. 3(c) show, this advantage in cost-reduction is very small. We can conclude that using the stationary densities as initial densities results in a sufficiently low cost. Also, it provides a good choice of initial density based on model parameters, because the stationary densities depend on local dynamics, which in turn depend on model parameters. The formulas for the stationary densities are included in the Supporting Information (Sections S1 and S2 in File S1).

We expand on the cost-minimizing property of stationary densities; we use them as initial densities throughout the rest of our study. We continue with the analysis of the critical cluster size’s dependence on model parameters. In particular, we focus on the value of sex ratio at birth (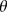), because both population stability and equilibrium density depend on this single parameter. For population stability and persistence, there is a range of permissible sex ratios, determined by the mortality rates (see Section S2 in File S1). Within this range, we define the optimal sex ratio 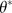 as the value maximizing the equilibrium population density [15], [17]:
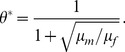
(17)


Note, that when the mortality rates are equal, the optimal sex ratio is 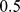, which is the parametrization, by definition, in the symmetric single-sex model.

**Figure 4:**
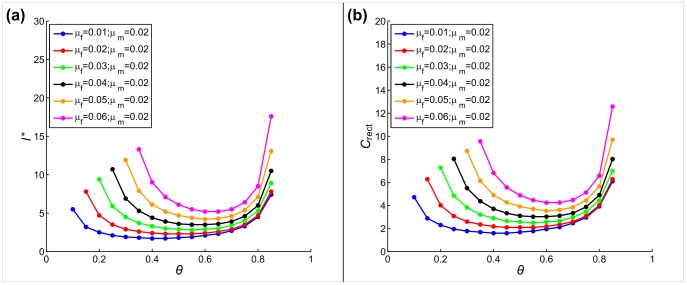
Scaling of (a) critical cluster size and (b) cost, with respect to sex ratio, at different mortality rate combinations. 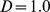
. Rectangular initial populations were used with stationary population densities.

We find that the smallest cluster size assuring restoration corresponds closely to the optimal sex ratio, and that any small deviation from the optimal value causes a small increase in the critical cluster size [Fig. 4(a)]. However, since the equilibrium population densities (serving as initial densities) decrease at suboptimal sex ratios (by definition; see Fig. S1 in File S1), the combined effect on the cost is non-trivial. As we see on Fig. 4(b), restoration cost is minimized at approximately the same sex ratios minimizing the critical cluster size, indicating that the cluster size is more sensitive to biased sex ratios than to equilibrium densities. We also find that strongly biased sex ratios approaching the boundary of the stability range cause both cluster size and restoration cost to diverge.

To complete the relationship between the sex ratio minimizing restoration cost (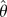) and the sex ratio that locally maximizes total equilibrium population density (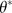), we compare the two quantities numerically. For the latter we have an analytical expression [Eq. (17)]. But our cost-minimizing sex ratios have only limited precision, since for each value of 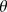 we employed binary search to determine the critical cluster size, which, in turn, determines the cost. Therefore we define a computational error bound on 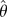 as the range of 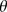 values that give critical cluster sizes within the error range of the minimum point’s cluster size found by binary search.

**Figure 5:**
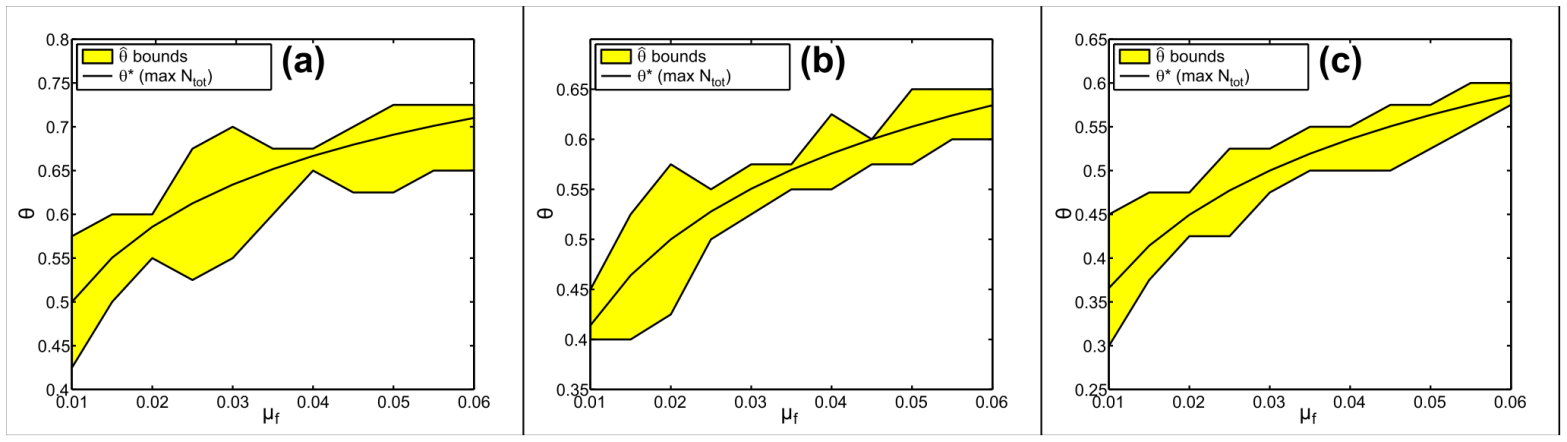
Comparision of density-maximizing and cost minimizing sex ratios. Density-maximizing sex ratio 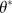 [Eq. (17)] and numerical bounds of cost-minimizing sex ratios 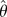 are calculated using rectangular population distributions with stationary initial densities, 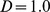, (a) 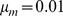, (b) 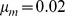, (c) 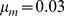.

Figure 5 offers comparison of the density-maximizing and cost-minimizing sex ratios. We can conclude that the sex ratio maximizing equilibrium density is identical to the sex ratio minimizing restoration cost, up to computational error.

To this point, our results reflect the assumption that individuals of each sex are introduced across the same extent of habitat. We now relax this constraint; that is, we permit 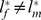, and ask whether the cost of restoration can be reduced by introducing individuals into sex-specific lengths of habitat. We denote the ratio of costs obtained by unequal and equal cluster sizes as:
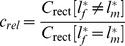
(18)


**Figure 6:**
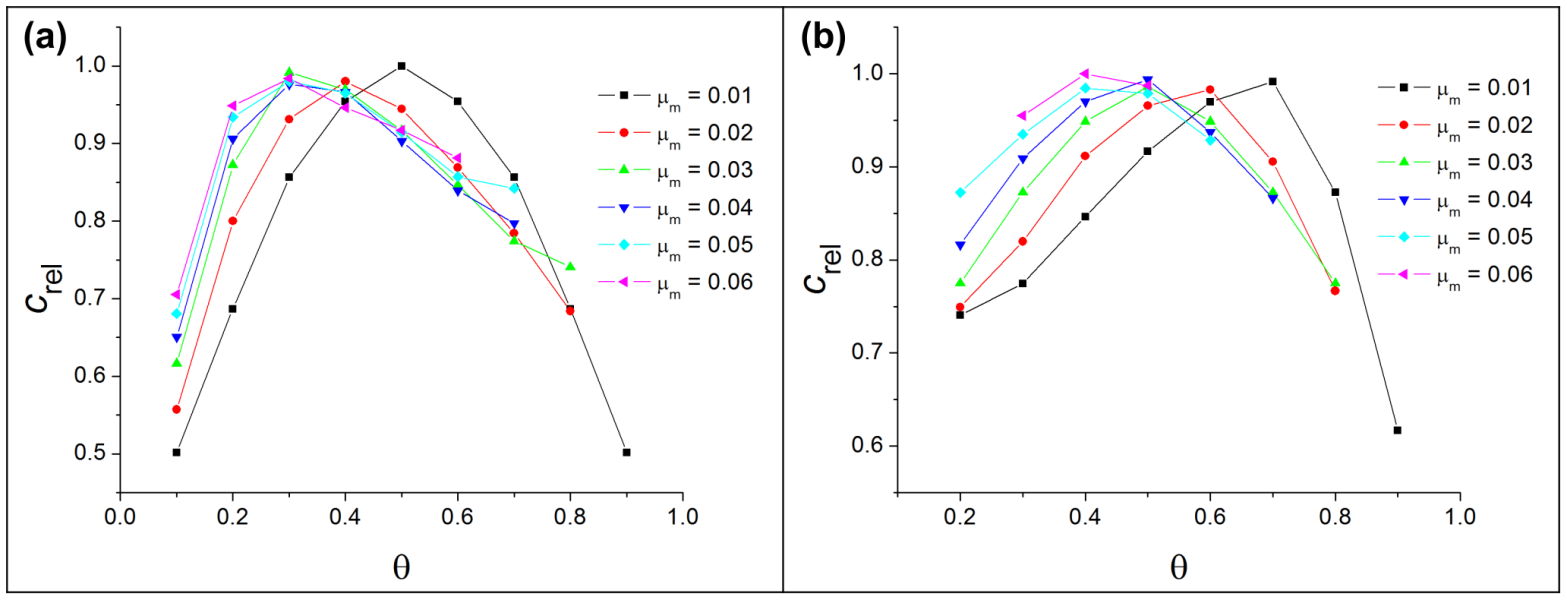
Minimum cost found by allowing different initial cluster sizes for males and females, relative to the case where cluster lengths are equal. Common parameter: 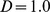. Individual parameters: (a) 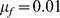, (b) 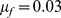.

Figure 6 presents our results. We find that optimality of the sex ratio plays a central role in reducing the cost of restoration. In particular, when the sex ratio equals the optimal (density-maximizing) value determined by mortality rates, the minimum cost is achieved with equally sized clusters. No further cost reduction can be achieved by allowing different cluster sizes for males and females. However, the relative advantage of sex-specific cluster size increases as the sex ratio deviates from the optimal value. If we consider that the absolute cost value diverges for strongly biased sex ratios [see Fig. 4(b)] we conclude that in such cases the savings achieved by adjusting the initial cluster sizes of each sex could be substantial.

### Simulated Annealing

We now relax all constraints on the initial cluster’s spatial distribution, and use simulated annealing to optimize sex-specific distribution shapes with respect to cost. At this stage we assume only that the cost-minimizing distributions have a finite support, and we carry out the minimization accordingly. However, the support of the function is allowed to grow or shrink by random shape changes during simulated annealing; see Supporting Information (Section S5 in File S1) for details.

**Figure 7:**
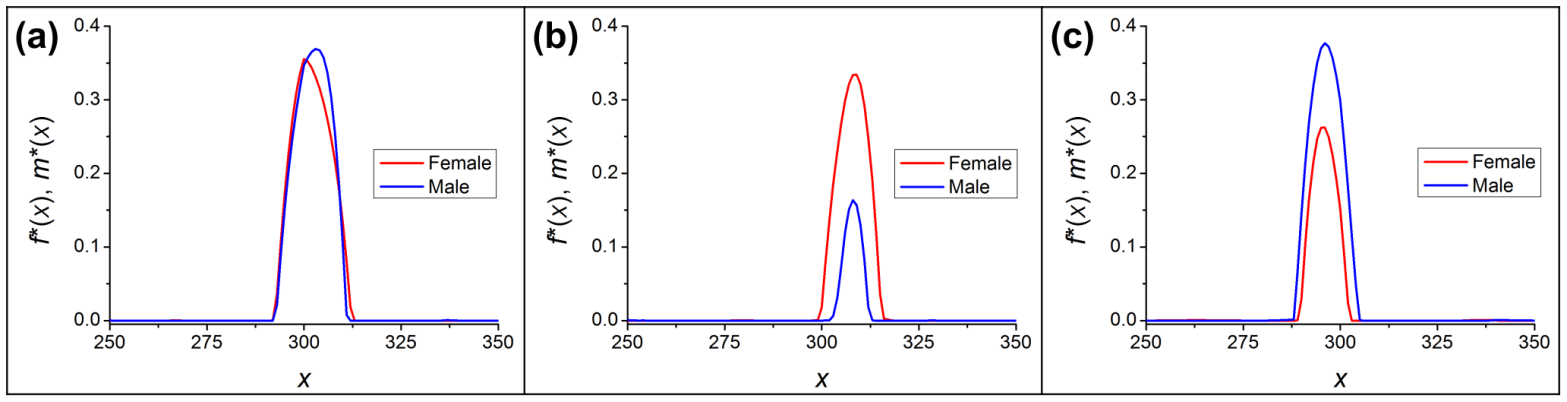
Shapes of initial density distributions that minimize cost, found by simulated annealing. The 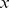 axis shows discretization grid coordinates; 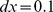 length unit per grid point. Parameters: (a) 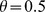, 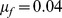, 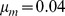, (b) 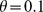, 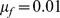, 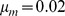, (c) 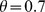, 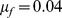, 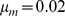.

By analyzing a series of minimum cost distributions obtained with simulated annealing, we observe the following. First, the distributions indeed have a finite support. Although we initialize them as such, the width of the optimal population distribution tends to become smaller, rather than larger, during simulated annealing. This effect during the minimization procedure is shown by Video S2; typical final, optimized shapes are shown in Fig. 7. It is also remarkable that the edges of the distributions go to zero very sharply; this property develops without any influence inherent to the procedure.

Generally, the final result is an “arch”-shaped distribution, with similar dimensions for females and males. Note that as the sex ratio diverges from its optimal value, we observe a change in the sizes of the two initial population distributions and in the height of the peaks. These changes in spatial distributions occur roughly in proportion to the system’s positive stationary densities [Fig. 7(b,c)]. Interestingly, the height of the peaks always falls between the stationary densities and the Allee threshold. Note that this shape provides the maximal rate of population growth possible during the first moments of the simulation, hence it combats the diffusion-amplified Allee effect most efficiently. Costs corresponding to the optimized distributions are denoted 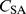 when we compare results with other methods.

## Discussion

We examined three approaches to minimizing the cost of a species’ restoration; the approaches differ in both ecological premises and mathematical methods. We considered the aperiodic, spatially inhomogeneous solution to the single-sex dynamics [Fig. 1(b)], critical cluster sizes of the rectangular initial setup [Fig. 4(a)], and simulated annealing of sex-specific initial distributions [Fig. 7].

**Figure 8:**
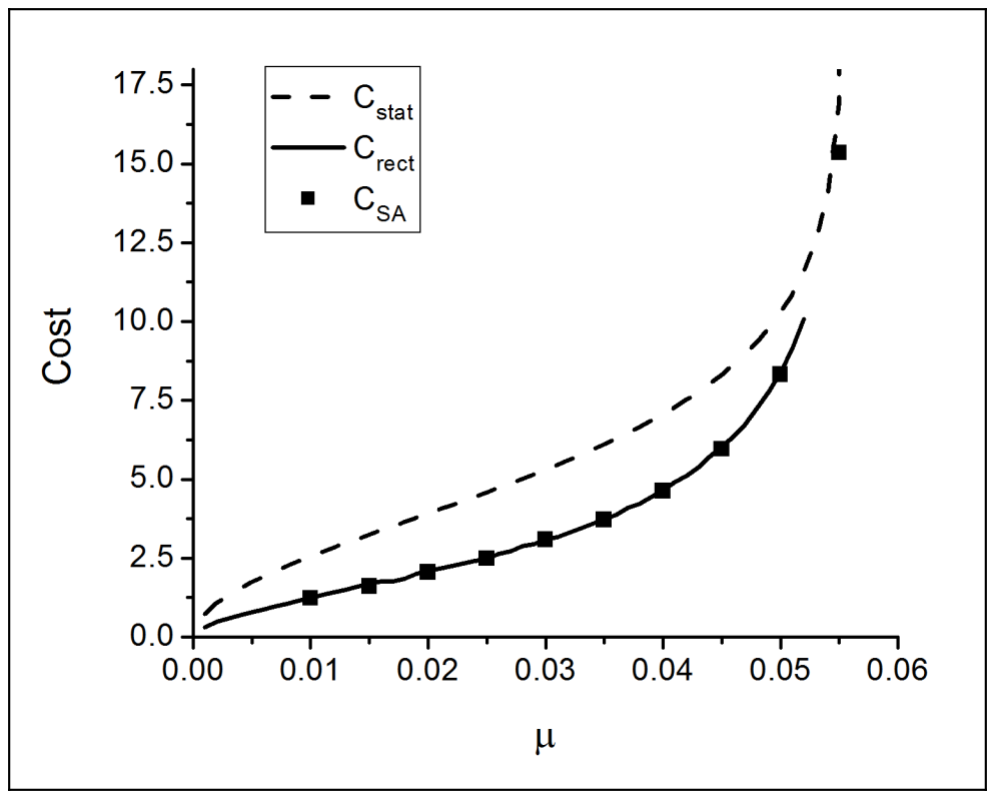
Comparison of minimum cost values in the symmetric model. Comparision includes cost values found by integrating the aperiodic stationary density, by using rectangular shape with stable stationary densities, and with simulated annealing.

Summary comparisons of the minimum restoration cost achieved by the different methods for the symmetric single sex model appear in Fig. 8. The aperiodic stationary solution to the dynamics gives significantly larger cost than the other approaches. Considering the shape of these distributions, they would likely prove difficult to implement in application. Restricting attention to the other two approaches, it is remarkable that simple rectangular distributions and the results of simulated annealing yield essentially identical costs.

**Figure 9:**
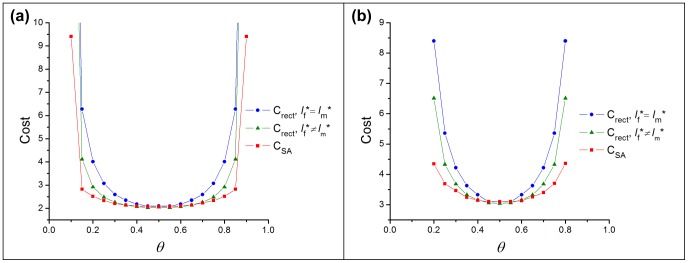
Comparision of minimum cost values in the two-sex model. Comparision includes cost values found by using rectangular shape with equal and unequal male and female cluster sizes, and by simulated annealing. (a) 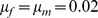, (b) 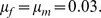

Figure 9 compares minimum costs for each critical-cluster analysis (i.e., a single cluster size and sex-specific cluster sizes) and costs incurred under simulated annealing. The critical cluster methods assume uniform initial population density within cluster bounds; simulated annealing lets initial densities depend on spatial location. The minimum cost varies little among methods as long as the sex ratio at birth does not deviate too much from the optimal value (here, 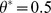). As sex-ratio bias increases, optimal sex-specific initial cluster sizes can lower the minimum cost of restoration. Simulated annealing reduces restoration cost even further, but this advantage becomes significant only at strongly biased (and biologically rare) sex ratios, and implementing such spatial distributions in application could prove difficult, negating any cost advantage. The same qualitative conclusions hold when we fix the sex ratio and increase the difference between the sexes’ respective mortality rates, because the mortality bias can also increase the difference between the optimal and any fixed sex ratio.

Our model assumes deterministic dynamics, which does not account for extinction due to demographic stochasticity in populations near an extinction threshold [43], [44]. This effect can be exaggerated when a population’s spatial dispersion leaves dynamically independent clusters near critical size [10], [45].

We assume diffusive dispersal. Many plants, and some animals, disperse only locally, i.e., the probability of long-distance dispersal is much lower than diffusion assumes [46]–[48]. Dispersal limitation becomes important when the number of discrete individuals is small [49], since random internal fluctuations can induce population extinction. Given discreteness and stochasticity, neither of which has a role in our cost-minimizing model, lattice-based results show that expected growth from rarity demands greater propagation, relative to mortality, as mean dispersal distance decreases [11], [50]. We also assume that no explicit interspecific interactions affect the population during restoration. Species occupying the community to be restored may facilitate restoration; for example, trees may attract birds that disperse seeds of other tree species [51]. Alternatively, resident species may resist the introduced species biotically [52], [53]. Interspecific interactions will often affect the likelihood of restoration success, as well as the cost. Consequences of these interactions can sometimes be expressed abstractly through the introduced species’ positive equilibrium density; in other cases, successful restoration may demand quantification of these interactions.

We assume an Allee effect arises from interaction of self-regulation with a birth rate that depends on the density of each sex. In the context of restoration, a two-sex dynamics may be essential to predicting spatial-expansion rate if dispersal differs between sexes [54]. We model mating encounters *via* mass-action, which should be reasonable for animals maintaining individual home ranges, or for dioecious plants with random mating. Alternative “marriage functions” [55] apply to certain species, particularly for polygynous or polyandrous mating systems.

We modeled a single species’ restoration only. Habitat restoration may attempt to manage particular multi-species interactions, or may seek to promote growth of many threatened species [1]. Our cost function ignores feedback of a species’ restoration on other biotic processes, or on economic stake-holders who incur post-restoration costs [56].

Our results suggest some considerations for species restoration. First, if a species disperses rapidly, individuals should be introduced concurrently, rather than serially. The initial population will increase only if the density exceeds any Allee threshold, and continues to do so as individuals disperse. Intuitively, the number/density of individuals introduced should increase with their dispersal rate.

Second, restoration cost declines little by introducing a species at a density below the (estimated) carrying capacity, unless the species disperses very slowly. Third, a rectangular spatial distribution adds little or no proportional cost over the ogive profile assumed in our simulated annealing method, as long as the sex ratio is close to optimal. Spatial uniformity will likely prove more practical for most animals. Given a uniform density close to the positive, stable equilibrium, restoration should focus on an initial population whose expanse exceeds the critical-cluster size, which (again) increases with dispersal rate.

Finally, adjusting frequencies of the sexes in an initial population may decrease the cost of successful restoration. Of course, if one sex always limits population growth, an excess of that sex promotes restoration. If population growth depends on the density of each sex, introducing the sexes at different densities, or with different cluster sizes, may prove advantageous. Sex ratio at birth may be unbiased, but mortality rates may differ between sexes, particularly during dispersal. Adjusting the sex ratio at introduction to match frequencies at positive equilibrium densities should promote successful restoration, and reduce its cost.

## Supporting Information

File S1 Includes derivations of fixed points of dynamics, stability conditions, and technical details of simulated annealing.
(PDF)

Video S1 Evolution of density distributions of populations during diffusive spreading, according to the single-sex model in one dimension. Multiple independent simulations are presented simultaneously, each having identical model parameters, except for initial cluster length, as indicated on the legend. The video clearly shows the presence of a critical cluster length; initial cluster sizes above this limit result in population persistence (successful restoration), smaller cluster sizes result in extinction (failed restoration).
(AVI)

Video S2Density distributions of males and females in one dimension during simulated annealing. Red color denotes females, blue denotes males. The horizontal orange and green lines show the stable stationary densities of local dynamics, for males and females, respectively. As time advances, the temperature-like control parameter is lowered, resulting in decreasing intensity of random fluctuations. Eventually, the cost-minimizing distribution shapes are obtained. Model parameters: 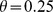, 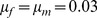, 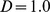.(AVI)
