# Supplementary material for: Restoration Ecology: Two-Sex Dynamics and Cost Minimization
Source: PLoS One. 2013 Oct 28;8(10):e77332. doi: 10.1371/journal.pone.0077332 (PMC3810464; doi:10.1371/journal.pone.0077332)
Supplement: File S1 — Includes derivations of fixed points of dynamics, stability conditions, and technical details of simulated annealing. (PDF) [file pone.0077332.s001.pdf]

Supporting Information

Restoration Ecology: Two-Sex Dynamics and Cost Minimization

F. Molnár Jr., C. Caragine, T. Caraco, G. Korniss

S1 Fixed points of the single-sex model

Recall the equation for the single sex model:

$$\partial_t u = D\nabla^2 u + \frac{1}{2}u^2(1-2u) - \mu u \tag{S1}$$

At equilibrium, the left hand side of the equation equals zero:

$$0 = D\nabla^2 u + \frac{1}{2}u^2(1-2u) - \mu u \tag{S2}$$

We have the following fixed points:

$$u_1^* = 0, \tag{S3}$$

$$u_{2,3}^* = \frac{1}{4} \mp \sqrt{\frac{1}{16} - \mu}. \tag{S4}$$

Subscript numbering of the fixed points matches the numbering of stationary-solution fixed points in the paper. u_1^* is the stable zero fixed point. u_2^* is unstable, u_3^* is stable, corresponding to the Allee threshold and positive stationary densities, respectively.

Since the discriminant in equation (S4) must be non-negative, we have a necessary condition for the existence of fixed points u_2^* and u_3^*:

$$\mu < \frac{1}{16}. \tag{S5}$$

S2 Fixed points of the two-sex model

For the two-sex model, our previous paper [1] provides derivations of the fixed points; we repeat them for the reader's convenience.

$$\begin{aligned} \partial_t f &= D_f\nabla^2 f + \theta\,(1-m-f)\,fm - \mu_{\mathrm{f}} f \\ \partial_t m &= D_m\nabla^2 m + (1-\theta)\,(1-m-f)\,fm - \mu_{\mathrm{m}} m\,. \end{aligned} \tag{S6}$$

We have a trivial fixed point at zero densities:

$$f_1^* = 0, \tag{S7}$$

$$m_1^* = 0. \tag{S8}$$

To obtain the non-trivial fixed points, we first manipulate the two stationary state equations, obtained from Eqs. (S6), to write a simple quadratic equation for the stationary total density,

$$N(1-N) = \frac{\mu_{\mathrm{f}}}{\theta} + \frac{\mu_{\mathrm{m}}}{1-\theta}\,, \tag{S9}$$

yielding solutions

$$N^{\pm} = \frac{1 \pm \sqrt{\mathbf{D}}}{2} \tag{S10}$$

with

$$\mathbf{D}(\mu_\mathrm{f}, \mu_\mathrm{m}, \theta) = 1 - 4\left(\frac{\mu_\mathrm{f}}{\theta} + \frac{\mu_\mathrm{m}}{1-\theta}\right) . \tag{S11}$$

Finally, for the non-trivial female and male densities at equilibrium, we have

$$(f^*, m^*)_{2,3} = \left(\frac{\mu_\mathrm{m}}{1-\theta} \cdot \frac{1}{1-N^\pm} , \frac{\mu_\mathrm{f}}{\theta} \cdot \frac{1}{1-N^\pm}\right) . \tag{S12}$$

For $\mathbf{D} \geq 0$ all three fixed points are real. The trivial (zero density) solution [Eq. (S8)] and the "+" solution [Eq. (S12)] are locally stable, separated by an unstable (saddle) fixed point, the "−" solution in Eq. (S12). The stability of these fixed points can be easily analyzed by linearizing Eqs. (S6). For $\mathbf{D} < 0$, however, only one biologically meaningful (real) fixed point exists, the zero-density solution [Eq. (S8)], and extinction is always stable.

The biological significance of the structure of the above solutions is two-fold [1]. First, for $\mathbf{D} > 0$, the system exhibits the Allee effect. Unless the (initial) population density is sufficiently high ($N(t=0) > N^-$), the population goes extinct [1,2]. Second, provided that $\sqrt{\mu_\mathrm{f}} + \sqrt{\mu_\mathrm{m}} < 1/2$, there is a finite interval $\theta_{\mathrm{c}1}(\mu_\mathrm{f}, \mu_\mathrm{m}) < \theta < \theta_{\mathrm{c}2}(\mu_\mathrm{f}, \mu_\mathrm{m})$, where $\mathbf{D}(\mu_\mathrm{f}, \mu_\mathrm{m}, \theta) > 0$, i.e., such that the population can persist at equilibrium. These boundaries are given by:

$$\theta_{\mathrm{c}1,2}(\mu_\mathrm{f}, \mu_\mathrm{m}) = \frac{(1 + 4\mu_\mathrm{f} - 4\mu_\mathrm{m}) \pm \sqrt{(1 + 4\mu_\mathrm{f} - 4\mu_\mathrm{m})^2 - 16\mu_\mathrm{f}}}{2} . \tag{S13}$$

Between the two critical points, at

$$\theta^* = \frac{1}{1 + \sqrt{\mu_\mathrm{m}/\mu_\mathrm{f}}} , \tag{S14}$$

total density exhibits a maximum at

$$N^{\max} = N^+(\theta^*) = \frac{1 + \sqrt{1 - 4(\sqrt{\mu_\mathrm{f}} + \sqrt{\mu_\mathrm{m}})^2}}{2} \tag{S15}$$

where the female to male density ratio is $f^*/m^* = \sqrt{\mu_\mathrm{m}/\mu_\mathrm{f}}$.

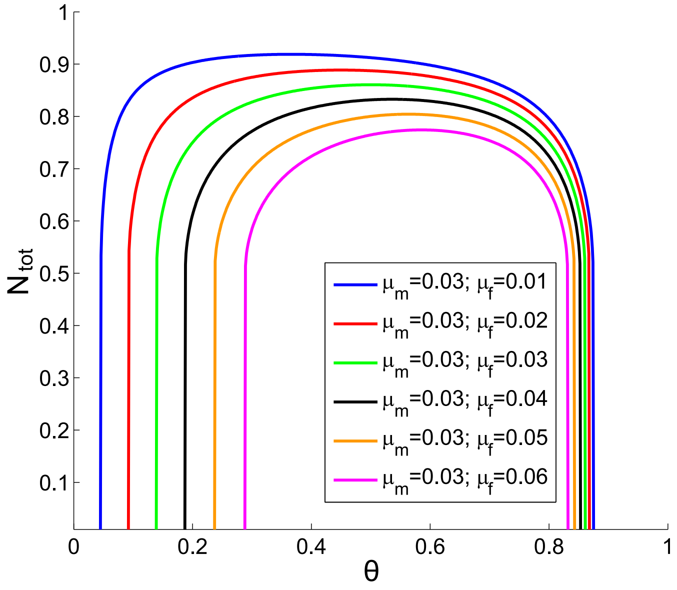

Figure S1: Total population density *vs* sex ratio, at various mortality rate combinations.

S3 Separatrices of stationary solutions for single-sex model

In the main text, we have derived an analytical expression for the relationship between density and its spatial derivative for the stationary solutions:

$$v(u) = \pm\sqrt{\frac{3u^4 - 2u^3 + 6\mu u^2 + 12DE}{6D}}. \tag{S16}$$

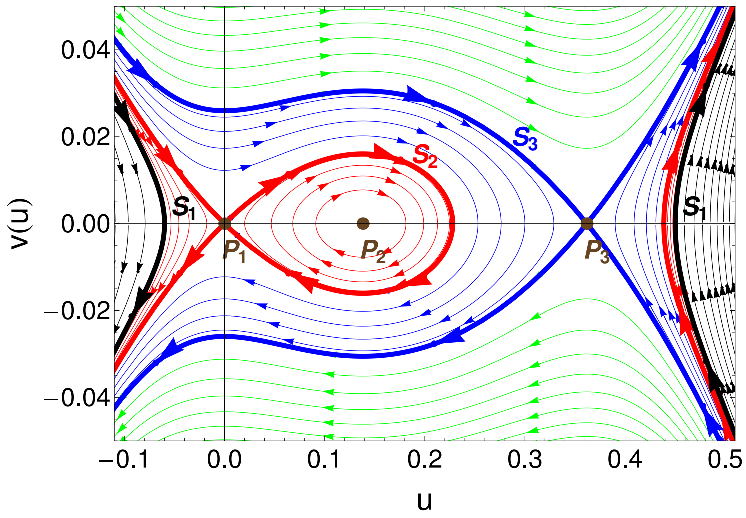

Figure S2: Phase plot of dynamics in the single-sex model, described by Eq. (S16). $D = 1.0$, $\mu = 0.05$. The dots indicate fixed points, the thick lines indicate separatrices. Different curves correspond to different E parameters, however, values of E were not chosen uniformly, for aesthetic reasons. This figure is also presented in the main text, it is repeated here for the reader's convenience.

Here we present further analysis to find the special values for E corresponding to separatrices, by analyzing local extremum points of $v(u)$. To find such points, we need only to study the fourth-order polynomial under the square root. Such a polynomial generally has three extrema. We utilize the phase plot [Fig. S2] to identify minima and maxima.

First, notice that when we are outside separatrix S_3 (green curves), $v(u)$ has a minimum at P_1, maximum at P_2 and minimum at P_3. The minimum at P_3 disappears when S_3 is reached. Therefore, we substitute the formula for u_3^* (the fixed point at P_3) into the polynomial under the square root in $v(u)$ and equate to zero:

$$3(u_3^*)^4 - 2(u_3^*)^3 + 6\mu(u_3^*)^2 + 12DE = 0, \tag{S17}$$

and we solve for E. We denote the result with the subscript of the corresponding separatrix:

$$E_3 = \frac{1}{24D}(u_3^*)^2(u_3^* - 6\mu) \tag{S18}$$

Therefore, if $E = E_3$, we obtain separatrix S_3.

Following the same logic, we find that the local minimum of $v(u)$ at P_1 becomes zero, when we reach separatrix S_2. We now substitute u_1^* (the fixed point at P_1) into the polynomial. Note that $u_1^* = 0$, which gives

$$12DE = 0. \tag{S19}$$

Therefore, we obtain separatrix S_2 when $E = E_2 = 0$. Further, in this case we can find the zero point values of $v(u)$ easily:

$$3u^4 - 2u^3 + 6\mu u^2 = 0. \tag{S20}$$

This gives the following zero points:

$$u_1 = 0, \tag{S21}$$

$$u_{2,3} = \frac{1}{3} \pm \sqrt{\frac{1}{9} - 2\mu}. \tag{S22}$$

This gives the necessary condition $\mu < \frac{1}{18}$ for the existence of S_2 and the corresponding aperiodic solution. Note, that this is a stricter condition than the existence of nonzero fixed points, defined by equation (S4).

Separatrix S_1 is found again using the same method. It corresponds to the case when the local maximum at P_2 becomes zero (and disappears). We substitute u_2^* into the polynomial to obtain:

$$3(u_2^*)^4 - 2(u_2^*)^3 + 6\mu(u_2^*)^2 + 12DE = 0 \tag{S23}$$

We solve for E and denote the result with the subscript number of the separatrix:

$$E_1 = \frac{1}{48D}(u_2^* - 2\mu)(u_2^* - 6\mu). \tag{S24}$$

Therefore, if $E = E_1$ we obtain separatrix S_1. Note that E_1 is negative. To summarize, the separatrices have corresponding E values that relate to each other as

$$E_1 < E_2 = 0 < E_3. \tag{S25}$$

S4 Analyzing stationary solutions of the single-sex model

Stationary solutions outsite S_2 separatrix (blue, green, and black curves on Fig. S2) have no physical or biological meaning, because they are unbounded, and extend to negative density ranges. Therefore we restrict our analysis to the periodic stationary solutions (red curves), and show that there is a minimum required spatial extent for their existence. In other words, we show that the period length cannot be smaller than a certain value.

In general, the length of period, L, specified by the value of E (note, $E_1 < E < E_2$ for periodic solutions) cannot be derived analytically, but we can find it numerically by integrating Eq. (S16) for given model parameters, see Fig. S3. As expected, L goes to infinity as E goes to E_2, that is, the solutions converge to S_2 separatrix, which corresponds to the aperiodic solution. It is also clearly visible that L is monotonically decreasing for smaller values of E. As E goes to E_1 the solutions converge to P_2 fixed point, which is a center. Since E cannot be smaller that E_1, the period length corresponding to E_1, denoted by L^*, is the minimum length. To find L^* explicitly, we need to analyze the asymptotic behavior of solutions around P_2. First, we find the stability matrix and its eigenvalues.

$$f(u,v) = \frac{\partial u}{\partial x} = v \tag{S26}$$

$$g(u,v) = \frac{\partial v}{\partial x} = \frac{-1}{D}\left(\frac{1}{2}u^2(1-2u) - \mu u\right) \tag{S27}$$

$$J = \begin{pmatrix} \frac{\partial f}{\partial u} & \frac{\partial f}{\partial v} \\ \frac{\partial g}{\partial u} & \frac{\partial g}{\partial v} \end{pmatrix} = \begin{pmatrix} 0 & 1 \\ \frac{3u^2 - u + \mu}{D} & 0 \end{pmatrix} \tag{S28}$$

We obtain the following eigenvalues:

$$\lambda_{1,2} = \pm\sqrt{\frac{3u^2 - u + \mu}{D}}. \tag{S29}$$

We are interested in the imaginary eigenvalues corresponding to the center (P_2):

$$\lambda_{P2} = \lambda_{1,2}\left(u_2^*\right) = \pm i\sqrt{\frac{16\mu - 1 + \sqrt{1-16\mu}}{8D}}. \tag{S30}$$

Asymptotically, as the periodic solutions approach P_2 they become circular orbits with an angular frequency given by the imaginary eigenvalue:

$$\omega = \sqrt{\frac{16\mu - 1 + \sqrt{1-16\mu}}{8D}}. \tag{S31}$$

From this we obtain the length of period (wavelength) in the limit of P_2:

$$L^* = \frac{2\pi}{\omega} = 2\pi\sqrt{\frac{8D}{16\mu - 1 + \sqrt{1-16\mu}}}. \tag{S32}$$

This is the minimum habitat size allowing periodic stationary solutions, corresponding to $E = E_1$.

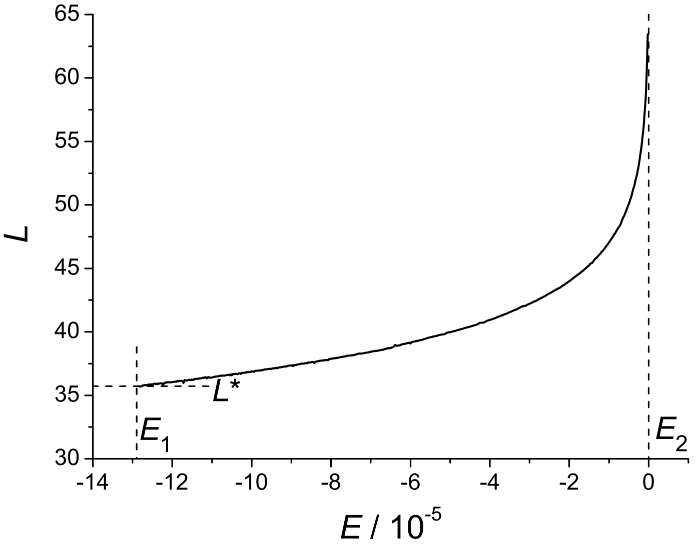

Figure S3: Length of period in periodic stationary solutions, found by numerical integration of $v(u) \equiv \mathrm{d}u/\mathrm{d}x$. $D = 1.0$, $\mu = 0.05$. L^* is the minimum habitat size allowing periodic stationary solutions.

S5 Simulated annealing

To move beyond the homogeneous distribution of the initial population as we seek to minimize cost, we need a method that finds the minimum cost in the domain of infinitely many possible distribution functions. The key to solving this problem is to define the initial distribution in discretized form. We can determine the eventual persistence or extinction of an initial population only by running the simulation to global equilibrium, and this simulation requires a spatial discretization for the integration. We use the same grid to discretize the initial distribution function. Further, we can assume that whatever initial distribution gives the minimum cost, it will have a finite support, therefore we can restrict the spatial extent of the distribution to a region in the center of the

simulated space. If this restricted spatial domain includes k grid points, we essentially reduce an infinitely detailed initial distribution to a k-dimensional function.

To find the minimum cost distribution, we use a global optimization method on the k-dimensional cost function, given the constraint that the population must persist. We use simulated annealing [4], which has the ability to explore the multidimensional domain randomly without getting stuck in local minima. It slowly guides itself to the global minimum of the given function (in our case, the cost function) by lowering a control parameter, which slowly lowers the expected function values the random moves can take. Eventually, a global minimum is found.

Simulated annealing requires an initial guess for the minimum-cost shape, and we must define a transition function that can transform any shape to any other in some finite number of iterations. At every step of the minimization, the new shape is proposed by applying the transition function to the previous shape. It is then accepted or rejected by the following criterion:

$$Pr(\text{accepted}) = \begin{cases} 1 & \text{if } \Delta C < 0 \\ \exp\left[\frac{-\Delta C}{T}\right] & \text{if } \Delta C \geq 0, \end{cases} \tag{S33}$$

where ΔC is the difference of cost between the newly proposed and previous functions, and T is a temperature-like control parameter that is lowered over time by a given "cooling schedule," so the probability of accepting changes that increase the cost function continuously declines. Eventually only changes that lower the cost are accepted, which finally moves the cost to a local minimum, but due to the gradual cooling and the stochastic nature of the procedure, this is also the global minimum with high probability.

Our minimization procedure starts with the following initial conditions:

$$z(x) := \max\left(0, H\left(1 - \frac{|(x - N/2)|}{W/2}\right)\right) \tag{S34}$$

$$T(0) := 0.1 \tag{S35}$$

Here, T is the temperature parameter and $z(x)$ denotes either male or female initial density distributions. The shape of $z(x)$ is an isosceles triangle standing on its shorter side of length W and having altitude H. These values are arbitrary, as long as the initial population they define generates a cost well above the sought minimum, so that they do not influence the minimization procedure. N denotes the width of simulated habitat; $W < N$. In our simulations, we used the following values: $N = 600$, $W = 100$ (measured in discretized grid points), $H = 0.4$, which is above the Allee threshold in every case we studied.

To minimize restoration cost with simulated annealing, we iterate the following steps. First, we determine the current spatial distribution's finite support:

$$a := \{x : z(x) > \xi \wedge \forall i < x : z(i) \leq \xi\} \tag{S36}$$

$$b := \{x : z(x) < \xi \wedge \forall i : a < i < b : z(i) > \xi\} \tag{S37}$$

$$\widetilde{a} := a - (b - a)/4 \tag{S38}$$

$$\widetilde{b} := b + (b - a)/4, \tag{S39}$$

where ξ is a cutoff threshold set to 10^{-3}. We use a randomized Gaussian function as transition function to generate a new proposed shape. The mean of this function is chosen from $[\widetilde{a}, \widetilde{b}]$ interval. Therefore, we allow for increasing the width of the density distributions, should the simulated annealing take that direction. The bell-shaped curve is defined by the following parameters:

$$M := \widetilde{a} + \alpha(\widetilde{b} - \widetilde{a}) \tag{S40}$$

$$A := 2\beta T \tag{S41}$$

$$V := (\widetilde{b} - \widetilde{a})/8 + 5\gamma T(\widetilde{b} - \widetilde{a}) \tag{S42}$$

$$S := \begin{cases} -1 & \text{if } \delta < 1/2 \\ 1 & \text{if } \delta \geq 1/2 \end{cases}, \tag{S43}$$

where M, A, V and S represent mean, amplitude, variance and sign, respectively, and α, β, γ and δ are uniform random numbers in the range $[0, 1)$. Note, that amplitude and variance also depend on the current temperature, T, which helps the minimization process by making smaller changes as T is reduced. The transition function is defined as

$$g(x) := \frac{SA}{\sqrt{2\pi V}} \exp\left[\frac{-(x-M)^2}{2V}\right]. \tag{S44}$$

We then add $g(x)$ to the current shape, resulting in a new proposed shape:

$$\widetilde{z}(x) := \max\left(0, z(x) + g(x)\right). \tag{S45}$$

We calculate shape distributions separately (independently) for both males and females. After the new shapes have been generated, the cost of the proposal is evaluated, and then accepted or rejected according to equation (S33). Finally, the temperature is lowered using a simple cooling schedule:

$$T(t+1) = 0.9999T(t). \tag{S46}$$

The iteration of these steps starts at $T(0) = 0.1$ and continues until $T(t) < 10^{-4}$.

The constraint requiring successful restoration must be checked by running the simulation until convergence to a homogenous stationary state, persistence or extinction. If the newly proposed shapes result in extinction, the shapes are always rejected regardless of Eq. (S33). Therefore, given that all previously accepted shapes resulted in survival, and given the convexity of the transition function (Gaussian), we know that when the newly proposed shapes increase the cost, then survival is guaranteed and there is no need to check it with a simulation. A test is only necessary when the cost is reduced. Still, this means that we need to run a numerical simulation for almost every second Monte Carlo step, which is computationally very intensive. To improve performance, we use GPGPU computation (using graphics processing units of video cards for general purpose computations) with CUDA [2]. Using GPUs can significantly improve the performance of PDE integration [3]. This technology lets us carry out each simulation within a fraction of a second, giving a total time for the simulated annealing in the order of a few hours.

References

[1] Molnár F Jr, Caraco T, Korniss G (2012) Extraordinary sex ratios: cultural effects on ecological consequences. PLoS ONE 7(8): e43364.

[2] NVIDIA Corp. (2013) CUDA C Programming Guide, http://docs.nvidia.com/cuda/cuda-c-programming-guide/. Accessed: 01 June 2013.

[3] Molnár F, Izsák F, Mészáros R, Lagzi I (2011) Simulation of reaction-diffusion processes in three dimensions using CUDA. Chemometr. Intell. Lab. 108: 76-85.

[4] Kirkpatrick S, Gelatt CD, Vecchi MP (1983) Optimization by simulated annealing. Science 220: 671-680.
